# Electricity forecasting on the individual household level enhanced based on activity patterns

**DOI:** 10.1371/journal.pone.0174098

**Published:** 2017-04-19

**Authors:** Krzysztof Gajowniczek, Tomasz Ząbkowski

**Affiliations:** Department of Informatics, Faculty of Applied Informatics and Mathematics, Warsaw University of Life Sciences, Warsaw, Poland; Chongqing University, CHINA

## Abstract

Leveraging smart metering solutions to support energy efficiency on the individual household level poses novel research challenges in monitoring usage and providing accurate load forecasting. Forecasting electricity usage is an especially important component that can provide intelligence to smart meters. In this paper, we propose an enhanced approach for load forecasting at the household level. The impacts of residents’ daily activities and appliance usages on the power consumption of the entire household are incorporated to improve the accuracy of the forecasting model. The contributions of this paper are threefold: (1) we addressed short-term electricity load forecasting for 24 hours ahead, not on the aggregate but on the individual household level, which fits into the Residential Power Load Forecasting (RPLF) methods; (2) for the forecasting, we utilized a household specific dataset of behaviors that influence power consumption, which was derived using segmentation and sequence mining algorithms; and (3) an extensive load forecasting study using different forecasting algorithms enhanced by the household activity patterns was undertaken.

## 1. Introduction and problem statement

Throughout the EU, there has been considerable interest in smarter electricity networks, where increased control over electricity supply and consumption is going to be achieved thanks to investments and improvements in new technologies such as Advanced Metering Infrastructure (AMI). Smart metering is part of this movement, and it is perceived as a necessary step to achieving EU energy policy goals by the year 2020, that is, to cut greenhouse gas emissions by 20%, to improve energy efficiency by 20% and to ensure that 20% of EU energy demand is supplied by renewable energy sources.

Smart metering systems are a part of micro-grid which includes a variety of operational and energy measures including smart appliances, renewable energy resources and energy efficient resources. One of the most challenging problems associated with operation of micro-grids is the optimal energy management of residential buildings with respect to multiple and often conflicting objectives [1]. Recently, attention is paid to smart grid vision and smart homes that can optimize energy consumption and lower electricity bills. Developing a smart home energy management system has become a common global priority to support the trend towards a more sustainable and reliable energy supply for smart grid as indicated in [2–5].

First, the new metering infrastructure is expected to ensure automated reading and billing based on actual usage. Second, by collecting high frequency consumption data, the system meets prerequisite for the implementation of cost reflective prices, varying based on the time of consumption. Third, these new metering systems are intended to contribute to reductions in the overall energy consumption by increasing the energy awareness of the users. One of the most important aspects of smart metering systems is to encourage users to use less electricity by being better informed about their consumption patterns. Forecasting usage provides customers with the possibility of linking current usage behaviors with future costs. Therefore, customers may benefit from forecasting solutions through greater understanding of their own energy consumption and their future projections, allowing them to better manage the costs of their usage. By making energy consumption and future projections more transparent, it would be easy to understand how much we are actually using and how it would affect our budget in the future. Of course, we should remember that technology alone will not be enough to change the way people consume energy, but it provides a method for using energy in a deliberate and conscious way. Therefore, we believe that our research fits into an attempt to generate value added for individual customers within the field of Residential Power Load Forecasting (RPLF) methods.

In this paper, we will study an approach to forecast the hourly electricity loads of individual consumers for 24 hours by taking into account historical electricity consumption and the household’s behavioral data. In particular, based on smart metering data, we aim to provide answers to the following research questions:

1. Is it possible to provide accurate load forecasting for 24 hours on the individual household level and to what extent?
2. Are the clustering and sequence recognition algorithms good tools for identifying patterns of household behavior?
3. Do the usage pattern variables of the household enhance the forecasting accuracy of individual consumer loads?
4. What kind of forecasting methods and algorithms are appropriate to address high volatility data?

The structure of the paper is organized as follows: a short literature review on similar problems is provided in section 2. In section 3, the approach to detect household activity patterns based on the Almanac of Minutely Power Dataset (AMPds) [6] is shown. The household specific behavioral data influencing power consumption are derived using the segmentation and sequence mining algorithms. Then, in section 4, a number of numerical experiments aimed to provide accurate 24-hour forecasts on the household level are presented. The scalability of the approach based on WikiEnergy data [7] gathered for 46 households is shown in section 5. Finally, section 6 concludes the paper.

## 2. Literature review of similar problems

The field of electricity load forecasting is mature with numerous approaches that have been proposed throughout the years. They have usually focused on system demand level forecasting, which has a load reaching tens of megawatts or gigawatts. A general overview of short-term load forecasting can be found in [8–9], and some more classic surveys are provided in [10] and [11]. Different methods have been proposed for forecasting the electric load demand in the last decades. Some of the most popular include time series analyses with the autoregressive integrated moving average (ARIMA) method [12], fuzzy logic [13], the neuro-fuzzy method [14], artificial neural networks (ANNs) [15–17], and support vector machines (SVMs) [18–19].

**Fig 1:**
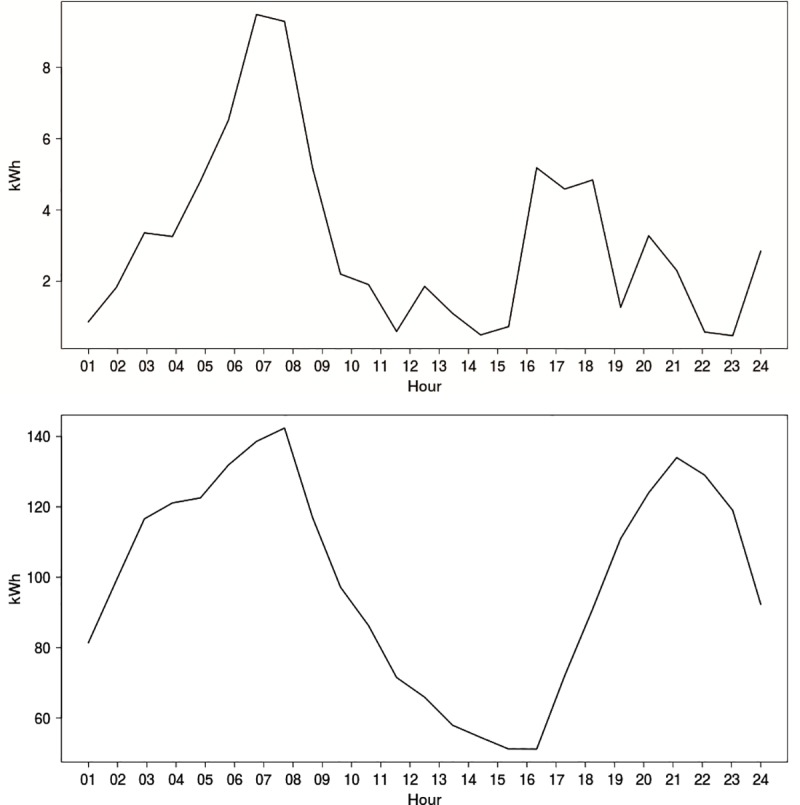
Daily electricity demand load profiles across a 24 hr period on 21st July 2013 based on WikiEnergy data [2]; (A) for an individual dwelling; (B) aggregated for 46 households.

Recently, with advances in communication infrastructure for remote and automated data reading, there has been increasing interest in RPLF. However, patterns of electricity use at a system demand level and at an individual level are very different. For instance, Fig 1A) shows the pattern of electricity use for a single random dwelling extracted from the WikiEnergy data [7]. The profile shows a peak in the morning at approximately 7–8 am and a second peak that is smaller than the peak in the morning between 4 pm and 6 pm. In contrast, Fig 1B) reflects a distinctly different pattern of electricity use for a group of 46 households on the same day of the year. The figure shows a smooth profile shape with relatively little electricity consumption in the early afternoon, a clearly defined peak in the morning and a slightly smaller defined peak in the evening.

Load forecasting on the individual household level is a challenging task due to the extreme system volatility as a result of dynamic processes composed of many individual components. Typical home loads are between 1 and 3 kWh and can be influenced by a number of factors, such as the operational characteristics of devices, the behaviors of the users, economic factors, time of the day, day of the week, holidays, weather conditions, geographic patterns and other random effects. Aggregation reduces the inherent variability in electricity consumption resulting in increasingly smooth load shapes, and as a result, the relative forecasting errors typically seen at the level of substations and power systems has been quite low in terms of MAPE (1% − 2%) [20], [15], [21]. The forecasting performance at the individual level shows much higher errors ranging from 20% to 100% (and even higher), and it depends on dwelling lifestyle and regularity of appliance usage [22–25].

Some interesting examples of electricity forecasting on the individual household level are indicated below.

Given sensor data collected from three residential homes, the authors of [26] aimed to determine which machine learning technique performed best at predicting whole building energy consumption for the next hour. The results showed that LS-SVM is the best technique for predicting each home’s future electrical consumption. In general, the proposed methods achieved MAPE of 1.60% to 13.41% for a 700 kWh commercial building and between 15% and 32% for three homes with mean consumptions close to 1.5 kWh.

In [27], the authors investigated high-resolution data from 3 private households collected over 30 days. The conclusion was that advanced forecasting methods can yield better forecasts, even when carried out on aggregated household consumption data that could be obtained from smart meters. Based on disaggregated data from smart homes, sensors with persistence and smart meter benchmarks reveal substantial forecast improvements of 4% to 33% in terms of the mean absolute error.

In [28], various methods were utilized to forecast peak demand for individual homes. The authors concluded that a home’s historic peak load and occupancy is a better predictor of peak load than temperature or season. They also showed that Seasonal Auto-Regressive Moving Average (SARMA) can be used to model both the intrinsic load pattern and consumer activity in a home and that it has 30% lower mean square error than regression-based techniques.

In [22], Kalman filter-based forecasting resulted in load forecast with MAPE of 30% for a sampling period and forecasting horizon equal to one hour. Shorter time intervals between receiving real-time measurement data from the customers' smart meter improved the accuracy of the proposed method and resulted in MAPE of nearly 13%.

In [29], based on power consumption measurements from 23 households collected across Japan, the authors proposed a support vector regression model and an activity sequence driven approach for inferring future activities and enhancing load forecasting. The activity sequence variable turned out to be an influencing factor that could improve the accuracy of load forecasting 15 minutes ahead for individual households, reaching 42% MAPE on average. Additionally, the study revealed that half of the households could not benefit from activity sequences to reduce their forecasting error.

In [30], the authors applied a number of forecasting methods including ARIMA, neural networks, and exponential smoothening using several strategies for training data selection (including day type and sliding window) with forecasting horizons ranging between 15 minutes and 24 hours. The evaluation was performed on two data sets; the first one was a single household in Germany, and the second one was for six households in the United States. The results indicate that forecasting accuracy varies significantly depending on the choice of forecasting method/strategy and the parameter configuration. In general, the MAPE ranged between 5% and greater than 100%, and the average MAPE for the first data set was approximately 30%, while it was approximately 85% for the other data set.

In [23], the authors presented an approach to forecast electricity loads on the individual household level using CART, SVM and MLP neural networks for 24 hour short-term load forecasts. The study concluded that a combination of historical usage data and household behavioral data could greatly enhance the forecasting of individual consumer loads. The obtained MAPE were 51% for the neural networks and 48% for the SVM.

Based on the literature review, there is a clear and increasingly recognizable research trend that looks at challenges associated with behavioral factors that impact the energy usages of individual appliances observed at the household level. The rationale is to provide feedback on usage patterns and derive significant underlying associations between several contextual factors including time of use and user activities. It is expected that the insights may increase awareness and understanding of home energy consumption and may be used as an additional variable that can enhance electricity forecasting.

## 3. Detecting household activity patterns

### 3.1. Data characteristics

The analysis was prepared based on the collection of electricity consumption data from a single house. The dataset is known as the Almanac of Minutely Power dataset (AMPds) [6] and contains two years of recorded energy consumption data (at one minute intervals) using 21 sub-meters and covering the time span between April 1st, 2012, and March 31st, 2014. The monitored house was built in 1955 in the greater Vancouver region in British Columbia, and it underwent major renovations in 2005 and 2006, which resulted in it receiving a Canadian Government EnerGuide rating of 82%.

The list of the monitored appliances is presented in Table 1. For the purpose of the numerical experiments, the cut-off value for each appliance was proposed based on visual analysis.

**Table 1:** Appliances monitored in AMPds.

Appliance	Cut-off value (Watts)
North Bedroom	2
Master and South Bedroom	9
Basement Plugs and Lights	7
Clothes Dryer	7
Clothes Washer	1
Dining Room Plugs	1
Dishwasher	1
Electronics Workbench	1
Security/Network Equipment	1
Kitchen Fridge	1
Forced Air Furnace: Fan and Thermostat	1
Garage	1
Heat Pump	100
Instant Hot Water Unit	7
Home Office	20
Outside Plug	1
Rental Suite Sub-Panel	50
Entertainment: TV, PVR, AMP	30
Utility Room Plug	8
Wall Oven	1

The analysis was narrowed to the most energy-intensive household appliances. These were Clothes Dryer (Dryer), Clothes Washer (Wash), Dishwasher (Dish), Heat Pump (Heat), and Instant Hot Water Unit (Instant). The other appliances were not considered due to their insignificant activities (e.g., Basement Plugs and Lights), continuous activity (e.g., Entertainment: TV, PVR, AMP), or those not showing any repetitive patterns (e.g., Electronics Workbench).

The starting point for the usage pattern detection was to prepare a matrix with switching on probabilities for each of the individual devices over a specified time period. The probabilities were estimated using the following formula.

$$P=\frac{Number\;of\;turn\;ON\;events\;in\;hour\;i}{Total\;number\;of\;turn\;ON\;events}.$$ (1)

Table 2 presents the matrix with observed probabilities for each appliance turn ON event over the analyzed period of 2 years. The probabilities for each appliance are equal to 1. The highest probabilities (more than 0.07) for each appliance are shown in bold.

**Table 2:** The matrix with the probabilities of appliance turn ON events in each hour.

hour	Clothes Dryer	Clothes Washer	Dishwasher	Heat Pump	Instant Hot Water Unit
**0**	0.017	0	0.017	0.016	0.006
**1**	0.002	0	0.005	0.028	0.001
**2**	0.007	0	0	0.035	0.001
**3**	0.006	0	0	0.038	0.001
**4**	0.008	0	0	0.043	0.001
**5**	0.008	0	0	0.047	0.005
**6**	0.008	0.002	0	0.063	0.04
**7**	0.013	0.008	0.005	**0.078**	0.059
**8**	0.01	0.042	0.016	0.061	0.05
**9**	0.021	**0.094**	0.028	0.054	0.054
**10**	0.044	**0.135**	0.05	0.053	0.044
**11**	0.05	**0.127**	0.056	0.054	0.036
**12**	**0.071**	**0.097**	0.045	0.043	0.037
**13**	**0.078**	**0.079**	0.052	0.041	0.038
**14**	**0.071**	**0.082**	0.051	0.041	0.034
**15**	0.06	0.061	0.053	0.051	0.051
**16**	0.065	0.057	0.057	0.052	0.068
**17**	0.063	0.05	0.056	0.033	**0.095**
**18**	0.051	0.044	**0.081**	0.029	**0.094**
**19**	0.07	0.054	**0.097**	0.029	**0.091**
**20**	0.05	0.044	**0.11**	0.025	**0.078**
**21**	0.049	0.02	**0.107**	0.035	0.055
**22**	**0.113**	0.004	**0.07**	0.039	0.039
**23**	0.065	0	0.044	0.012	0.022

It can be noticed that the highest probabilities to use the cloth dryer are at midday (between 12 am and 2 pm) and in the evening (10 pm). The clothes washer is used frequently between 9 am and 2 pm. The use of the dishwasher usually occur in the evening between 6 pm and 10 pm. The heat pump operates more frequently in the morning at 7 am, while the instant hot water unit reaches its peak in the evening, between 5 pm and 8 pm.

In the same manner, a larger table (S1 Table) has been created, which consists of 24 rows (representing hours) and 35 columns (representing appliances over the seven days of the week). For each appliance, seven columns show the probabilities of the turn ON events on a specified day of the week. In this case, the probabilities over the whole week are equal to 1, for each appliance.

### 3.2. Activity segmentation

In the industry, segmentation has been used by large electricity suppliers to group customers together that share similar characteristics in terms of electricity usage. However, its use at the individual household level has been somewhat limited. Currently, thanks to smart meter data, there is an opportunity to capture and present individual patterns of energy consumption derived by the clustering algorithms. The discovered patterns of home appliance usage can be visualized to help the users understand their own energy consumptions, and these patterns can be used to feed the forecasting models, which is our motivation. We believe such an enhanced set of explanatory variables can significantly improve the accuracy of the forecasts generated at the household level.

For this purpose, we propose **hierarchical clustering** and **grade data analysis**.

**Hierarchical cluster** analysis is an algorithmic approach to find discrete groups with varying degrees of similarity in a dataset represented by a similarity matrix. These groups are hierarchically organized as the algorithms proceed and may be presented as a dendrogram.

One of the most popular agglomerative clustering algorithm is Ward’s method [31]. Basically, it looks at cluster analysis as an analysis of variance problem, instead of using distance metrics or measures of association. It looks for groups of leaves, which it forms into branches. Then, the branches are formed into limbs and eventually into the trunk. Ward’s method starts out with n clusters of size 1 and continues until all of the observations are included in one cluster.

**Fig 2:**
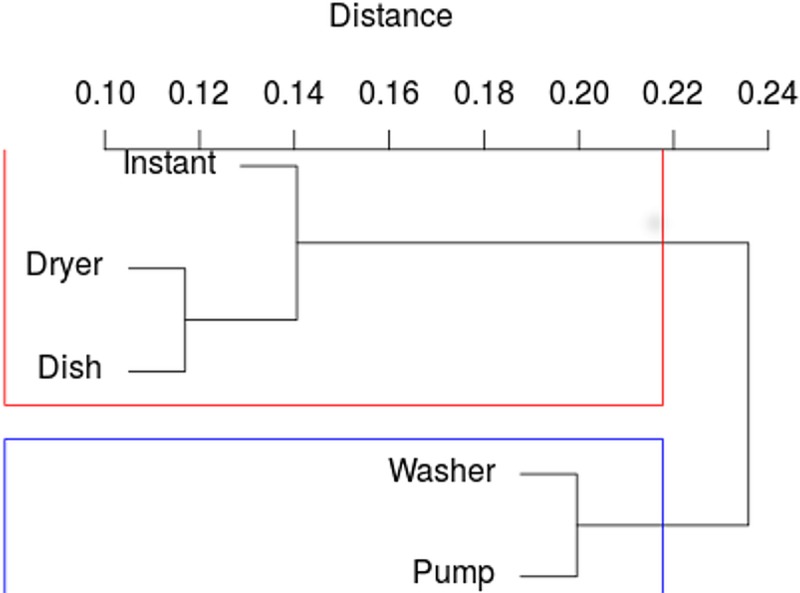
Dendrogram for grouping the electrical appliances throughout the day.

The purpose of this analysis is to discover similar profiles or, in other words, appliances with similar switch ON probability distributions throughout the day or the week. As a result of grouping using Ward’s method with the Euclidean distance measure, the following dendrogram for the data presented in Table 2 was obtained and presented in Fig 2.

The height of each edge of the dendrogram is proportional to the distance between the joined groups. As shown in Fig 2, there are two groups that are distinctly separated from each other. From the visual analysis of the dendrogram, it can be observed that the switch ON probabilities of the clothes washer and heat pump are very similar (cluster marked in blue). A similar correlation in periods of joint work can be seen in the case of the dishwasher, clothes dryer and instant hot water unit (clusters marked in red).

**Fig 3:**
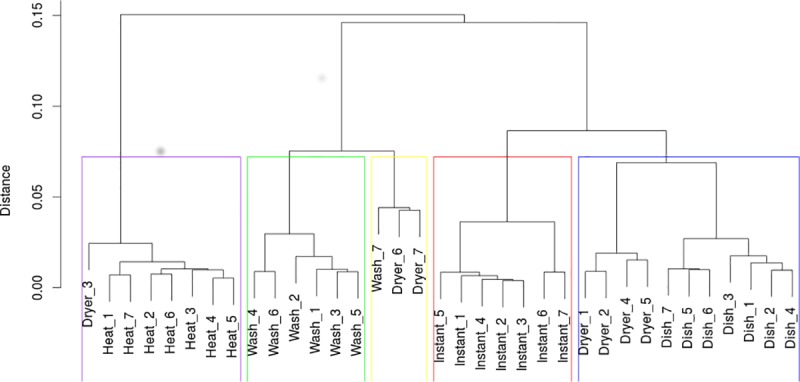
Dendrogram for grouping the electrical appliances throughout the week.

Graphical representation of the data from S1 Table is shown in Fig 3.

In the case of those data, it is difficult to draw clear conclusions about the dependencies of the co-occurrences of the appliances by day of the week. However, at the bottom, there is a cluster associated with the dishwasher activity on all of the week days (cluster marked in gray). Another group (marked in green) represents the washing machine over the entire week except Sunday. The usage profile of this device is most similar to the usage profile of the clothes dryer on the weekends (yellow). Finally, the profiles of the heat pump over the entire week are clustered in one group (on top of the chart, in purple). They are the least similar to the profiles of the other devices.

**Grade data analysis** is an efficient technique that works on variables measured on any measurement scales (including categorical) because it is based on dissimilarity measures such as concentration curves and some precisely defined measure of monotonic dependence. Its main framework is composed of grade transformation proposed by [32]. The idea is to transform any distribution of two variables into a convenient form of the so called grade distribution. This transformation leaves the orders of the variables, ranks, and values of monotone dependence measures (such as Spearman’s ρ^*^ and Kendall’s *τ*) unchanged. In case of empirical data, this approach is focused on analyzing the two-way table with objects/variables, which is preceded by proper recoding of variable values.

The main tool of the grade data methods is Grade Correspondence Analysis (GCA), which refers to classical correspondence analysis and extends it by the mean of the grade transformation. Briefly, GCA orders the variables/objects in a table in a way such that neighboring objects are more similar than those that are further apart, and at the same time, neighboring variables are also more similar than those that are further apart. After the optimal ordering is found, it is possible to aggregate neighboring objects and neighboring variables and, therefore, to build segments with similar distributions.

The data structure presented in Table 2 has been analyzed using the GradeStat tool [33], which was developed at the Institute of Computer Science Polish Academy of Science.

The first step was to calculate over-representation ratios for each field (cell) of the table. A given *m* × *k* data matrix with non-negative values can be visualized using an over-representation map in the same way as a contingency table [34]. Instead of frequency *n*_*ij*_, the value of the j-th variable for the i-th object is used. Next, it is compared in a contingency table with the corresponding neutral or fair representation *n*_*i•*_ × *n*_*•j*_/∑∑*n*_*ij*_ where *n*_*i•*_/∑_*j*_ *n*_*ij*_, *n*_*•j*_/∑_*i*_ *n*_*ij*_. The ratio of the first and second expressions is called the over-representation ratio. An over-representation surface over a unit square is divided into *m* × *k* rectangles situated in m rows and k columns, and the area of the rectangle placed in row i and column j being equal to fair representation of normalized *n*_*ij*_. For instance, taking into account the use of the dishwasher at 8 pm, the ratio would be equal to 1.79153 because the probability of using the dishwasher in this hour is 0.11 and the row sum is 0.307 (for five appliances). Thus, we have 1.79153 = 0.11/(0.307/5). Using the over-representation ratios, the over-representation map for the initial raw data can be constructed. The color of each field in the map depends on the comparison of two values: (1) the real value of the measure connected to the considered field and corresponding to the population element; (2) the expected value of the measure.

**Fig 4:**
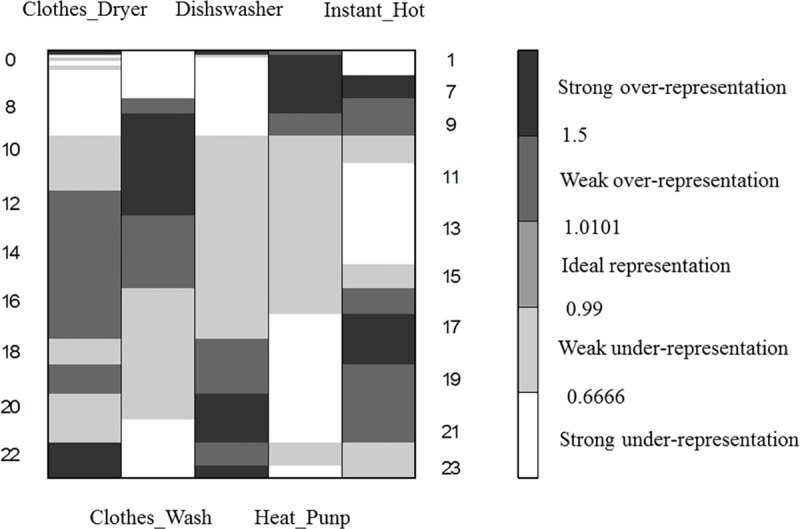
The initial over-representation map.

Fig 4 presents the initial over-representation map for the analyzed data. The colors of the cells in the map are grouped into three classes:

1. *gray*–the measure for the element is **neutral** (ranging between 0.99 and 1.01). which means that the real value of the measure is equal to its expected value;
2. *black* or *dark gray*–the measure for the element is **over-represented** (between 1.01 and 1.5 for weak over-representation and more than 1.5 for strong), which means that the real value of the measure is greater than the expected one;
3. *light gray* or *white* the measure for the element is **under-represented** (between 0.66 and 0.99 for weak under-representation and less than 0.66 for strong under-representation). which means that the real value of the measure is less than the expected one.

The next step was to apply the grade analysis to measure the dissimilarity between two data distributions in order to reveal the structural trends in the data. For this reason. Spearman's ρ^*^ was used as the total diversity index. The value of ρ^*^ strongly depends on the mutual order of the map’s rows and columns. To calculate ρ^*^ the concentration indices of differentiation between the distributions are used. The basic procedure of GCA is executed through permuting the rows and columns of a table in order to maximize the value of ρ^*^. After each sorting, the ρ^*^ value increases, and the map becomes more similar to the ideal one, which, means that the darkest fields are placed in the upper-left and lower-right map corners while the rest of the fields are assigned according to the following property: the farther from the diagonal towards the two other map corners (the lower-left and the upper-right), the lighter gray (or white) the field.

**Fig 5:**
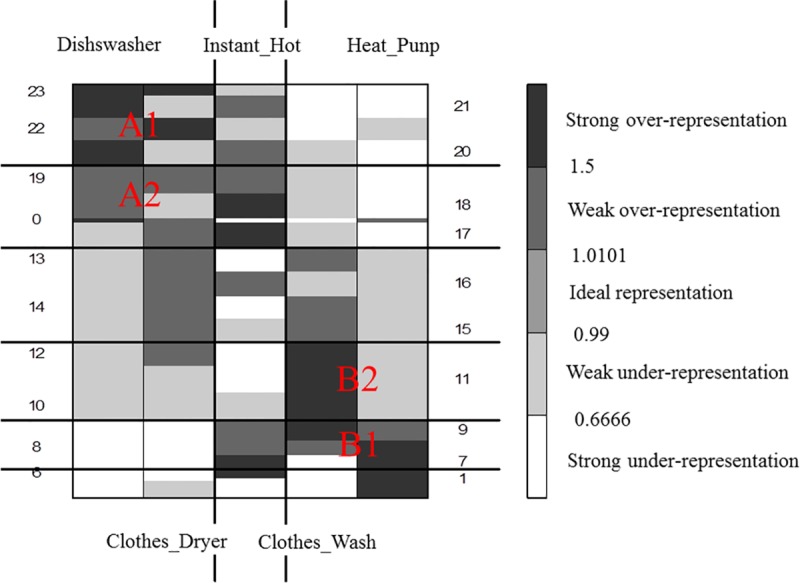
The final GCA over-representation map with clusters.

The result of the GCA procedure is presented in Fig 5. Additionally, cluster analysis was performed through the aggregation of some columns into one column (and for the rows respectively). The optimal number of clusters is obtained when the changes of the subsequent ρ^*^ values appear to be negligible as referenced in [35].

The resulting order presents the structure of the underlying trends in the data. The clusters show typical usage patterns of home appliances. Two clusters in the top left corner (marked A1 and A2) are similar in terms of the same usage hours; they represent the dishwasher and the clothes dryer. This group of appliances is strongly over-represented in the evening hours, whereas they are strongly under-represented in the morning. The clusters in the bottom right corner are related to the heat pump and the clothes washer. The usages of these appliances usually occurred in the morning or at midday (marked with B1 and B2). The profile of the water heating device (located in the center of the over-representation map) is not related to the usages of the other appliances.

**Fig 6:**
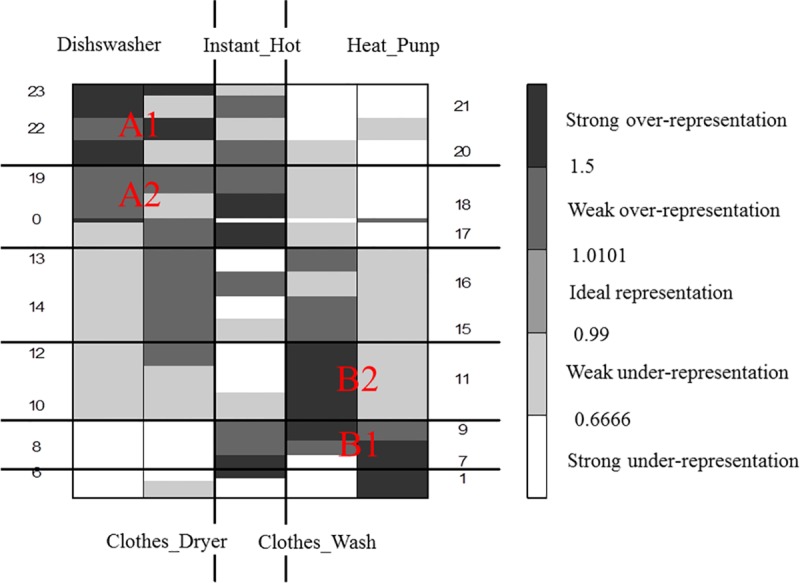
The final GCA over-representation map including appliances and the days of their usage.

Additional, separate analysis was performed using the data from S1 Table. This was to inspect the differences in household usage patterns between weekdays, please see Fig 6 for the results.

The analysis revealed that the usage profiles for the devices broken down by day of the week differ only slightly from those observed as a whole. Only some cases, e.g. clothes dryer on Wednesday (marked with red border), are outside their natural group. In general, the patterns of behavior are similar to those presented in Fig 5.

### 3.3. Activity sequence mining

Sequence mining is an exploration technique that focuses on discovering statistically relevant patterns in the form of a sequence for a given data set. The resulting rules (patterns) adopt the following form of conditional statements: if appliance A was used, then appliance B will be used next.

In the home energy research field, sequence mining can be applied to capture the use of appliances in sequence. This kind of analysis gives insight that can help understand how power consumption is influenced by certain activities and their sequences and how those activities are related to each other.

Within the collected data, it is possible to track sequences of different activities that a household performs throughout a day. To understand the daily activity sequences of a household, a sequence is given by all of the activities performed by the household during the day, ordered by the start time of the activity.

To discover only interesting and valuable rules from the dataset, three basic measures were used for their evaluation [36]: (1) support is defined as the proportion of days containing a specific itemset of the appliances to all of the days; (2) confidence is defined as the proportion of the observed support of the specific rule to the support of the left side item (corresponds to the conditional probability denoting if the left side occurred then also with some probability the right side of rule will occur); (3) lift is defined as the proportion of the observed support of the rule to the product of the supports of both sides of the rule, and it shows, in business terms, how many times more likely the appearance of appliance B with appliance A is than that with any other randomly chosen appliance. These measures are calculated according to following formulas:

$$support(appliance\;A\to appliance\;B)=\frac{\left|appliance\;A\to appliance\;B\right|}{\left|all\;days\right|}.$$ (2)

$$confidence(appliance\;A\to appliance\;B)=\frac{supp(appliance\;A\to appliance\;B)}{supp(appliance\;A)}.$$ (3)

$$lift(appliance\;A\to appliance\;B)=\frac{supp(appliance\;A\to appliance\;B)}{supp(appliance\;A)∙supp(appliance\;B)}.$$ (4)

The goal of sequence rules is to find all of the strong rules, such that support level and confidence level are greater than a minimum threshold value.

By considering only the rules with support greater than 1%, a minimum difference between the appliances’ switch on events of at least 1 hour, and a maximum difference between consecutive elements in the sequence, the usage patterns presented in Table 3 were discovered.

**Table 3:** Selected sequential rules extracted from AMPDs data.

Sequence rule	Support (%)	Confidence (%)	Lift
instant & clothes washer & dishwasher = = > clothes washer & dishwasher	4	80	13.86
instant & clothes washer = = > dryer & dishwasher = = > dryer & dishwasher	3	56	9.77
heat & clothes washer = = > instant & heat & clothes washer = = > heat & dryer	3	67	6.68
instant & heat & clothes washer = = > instant & heat & clothes washer = = > heat & dryer	3	64	6.40
instant = = > heat & clothes washer = = > instant & heat & dryer	5	47	5.62

All of the observed sequential rules have lift greater than one, which means that the occurrence of the elements on the left side of the rules influences the occurrence of the elements contained on the right side of the sequential rule. In particular, the following behavior patterns were observed:

- with support equal to 4% and a confidence of 80%, if in a given hour the instant unit & clothes washer & dishwasher have been used, then in the next hours, the clothes washer & dishwasher will also have been used;
- with support equal to 3% and a confidence of 56%, if in a given hour the instant unit & clothes washer have been used, then in the next hours, we could expect the dryer & dishwasher to be switched on, and they could operate for the next hour;
- with support equal to 3% and a confidence of 67%, if in a given hour the heat pump & clothes washer have been used, then in the next hours, the instant unit & heat pump & clothes washer will also have been used and followed by the heat pump & dryer;
- with support equal to 3% and a confidence of 64%, if in a given hour the instant unit & heat pump & clothes washer have been used, then in the next hours, the same set of appliances will also have been used and followed by the heat pump & dryer;
- finally, with support equal to 5% and a confidence of 47%, if in a given hour the instant unit was switched on, then in the next hours, the heat pump & clothes washer will also have been used and followed by the instant unit & heat pump & dryer.

## 4. Forecasting experiments

### 4.1. Accuracy measures

To assess the model performance for forecasting, three measures were used. These were precision, resistant mean absolute percentage error and accuracy [37].

Precision is defined as the measure of how close the model is able to forecast the actual load. To measure precision, the mean squared error (MSE) was used:

$$MSE=\frac{1}{n}\sum_{i=1}^{n}{\left(W_{hi}-P_{hi}\right)}^{2}.$$ (5)

where *W*_*hi*_ is the observed load in hour *i* and *P*_*hi*_ is the forecasted load in hour *i*.

Mean Absolute Percentage Error (MAPE) was the second measure that was used, This measure satisfies the criteria of reliability. ease of interpretation and clarity of presentation. However, it does not meet the validity criterion because the distribution of the absolute percentage errors is usually skewed to the right due to the presence of outlier values. In these cases, MAPE can be highly over-influenced by some very bad instances and can disrupt quite good forecasts. Therefore, we propose an alternative measure, called resistant MAPE or r-MAPE. based on the calculation of the Huber M-estimator, which helps to overcome the aforementioned limitation [38].

An M-estimator for the location parameter *μ* using the maximum likelihood (ML)-estimator is defined as a solution *θ* to

$$\sum_{i=1}^{n}\rho (\frac{|\frac{W_{hi}-P_{hi}}{W_{hi}}|-\theta }{\sigma })=\underset{\theta }{min}.$$ (6)

or

$$\sum_{i=1}^{n}\varphi (\frac{|\frac{W_{hi}-P_{hi}}{W_{hi}}|-\theta }{\sigma })=0.$$ (7)

where *φ* = *ρ*′.*σ* is the scale parameter. For a given positive constant *k*, the Huber [39] estimator is defined by the following function in *φ* (3)

$$\varphi (k)=\left\{ \begin{array}{l} k\;x>k\\ x\;-k\leq x\leq k\\ -k\;x<k \end{array}\right..$$ (8)

where *k* is a tuning constant determining the degree of robustness and set at 1.5. The function is known as metric Winsorizing and brings in extreme observations to *μ* ∓ *k*. In practice, *σ* is not known, thus, a MAD robust estimator was used:

$$MAD=median(\left|x_{i}-median(x_{i})\right|).$$ (9)

Finally, an accuracy measure was used, which identifies how many correct forecasts the model makes, where the term correctness is defined by the user. This can be done by defining correct forecasts as values within a percentage range of the actual load. However. for low loads, a percentage range may become insignificant. For a load of 0.1 kWh, a 15% range would be 0.085–0.115, and a forecast of 0.2 kWh will be considered wrong, but in practice, such a forecast would be acceptable. To address this false loss of accuracy, we set two scales to measure the accuracy. In this study, we set a 15% range of error for accuracy, but if the load was smaller than 1 kWh, then we considered the range of ±0.15 kWh as the range of acceptable forecasts. Therefore, accuracy for hour i was given as:

$$Accuracy=\sum \;1\{W_{hi}>1\&|W_{hi}-P_{hi}|<P_{hi}*0.15\}+\sum \;1\{W_{hi}.1\&|W_{hi}-P_{hi}|<0.15\}.$$ (10)

### 4.2. Predictors

In this research, we focused on forecasting the electricity usage of a particular household for 24 hours ahead. To forecast the load, we constructed a feature vector with attributes as presented in Table 4. The attributes were constructed based on time series with hourly electricity demand. Additionally, other variables were collected, including temperature, humidity, and date.

**Table 4:** Feature vector used in forecasting.

Attribute No.	Description	Formula
1–24	Hour indicator (dummy variable)	*G*_*i*_, *i* = 1,…,24
25–55	Day of the month indicator (dummy variable)	*D*_*i*_, *i* = 1,…,31
56–62	Day of the week indicator (dummy variable)	*T*_*i*_, *i* = 1,…,7
63–74	Month indicator (dummy variable)	*M*_*i*_, *i* = 1,…,12
75	Holiday indicator (dummy variable)	*S*
76	Sunset indicator (dummy variable)	*N*
77–100	Load of previous 24 hours	*Z*_*g*−*i*_, *i* = 1,…,24
101–104	Minimum load of previous 3, 6, 12, 24 hours	*min*{*Z*_*g*−1_,…,*Z*_*g−i*_}. *i* = 3,6,12,24
105–108	Maximum load of previous 3, 6, 12, 24 hours	*max*{*Z*_*g*−1_,…,*Z*_*g−i*_}. *i* = 3,6,12,24
109–114	Load in the same hour of the previous week (6 days)	*Z*_*g*,*d−i*_, *i* = 2,…,7
115–118	Load in the same hour of the same day in previous weeks (4 weeks)	*Z*_*g*,*d−i*_, *i* = 14,21,28,35
119–122	Average temperature observed over previous hourly periods	*avg*{*T*_*g−i*_,…,*T*_*g*−*i*[+1]_} *i* = 1,3,6,12,24
123–128	Average temperature observed in the same hour over the previous week (6 days)	*T*_*g*,*d−i*_, *i* = 2,…,7
129–132	Average weekly temperature observed in previous i-day periods	*avg*{*T*_*g*,*d−i*_,…,*T*_*g*,*d*−*i*[+1]_}. *i* = 7,14,21,28,35
133–136	Average humidity observed over previous hourly periods	*avg*{*W*_*g−i*_,…,*W*_*g*−*i*[+1]_}. *i* = 1,3,6,12,24
137–142	Average humidity observed in the same hour over the previous week (6 days)	*W*_*g*,*d−i*_, *i* = 2,…,7
143–146	Average humidity observed in previous i-day periods	*avg*{*W*_*g*,*d−i*_,…,*W*_*g*,*d*−*i*[+1]_}. *i* = 7,14,21,28,35

Notation [+1] stands for the next element from the set of indices i {1,3,6,12,24} e.g. *avg*{*T*_*g*,*d*−1_,…,*T*_*g*,*d*−3_} or *avg*{*T*_*g*,*d*−3_,…,*T*_*g*,*d*−6_}.

Electricity demand varies over time depending on the time of day (daily cycles), day of the week (weekly cycles), day of the month (monthly cycles), season (seasonal cycles) and occurrence of holidays. Therefore, we enriched the analysis with an additional 76 dummy variables that described the hour (1–24), 31 variables associated with the day of the month, 7 variables associated with the day of the week, 12 variables associated with the month, one variable indicating a holiday and one variable indicating the sunset in a particular hour.

The main variables taken into account in the forecasting process are those derived directly from the time series. The features were created by the decomposition of the time series, and they define, among others, minimum, maximum and actual demand at certain intervals (up to 35 days back).

In addition to the attributes that describe the historical electricity consumption, a set of behavioral features describing the habits of the household was prepared. Those are associated with the use of certain electrical appliances as presented in Table 5. The presented set of attributes describes the household behavior patterns that were discovered during the earlier stages of the research with the method of segmentation and sequence analysis.

**Table 5:** Feature vector to describe hourly usage patterns.

Attribute No.	Description	Formula
147–166	Number of switch on states (activations) for each appliance (Dryer, Wash, Dish, Heat, Instant) over previous hourly periods	∑_*ON*_{Appliance_*g−i*_,…,Appliance_*g*−*i*[+1]_}, *i* = 1,3,6,12,24
167–176	Number of switch on states (activations) for each appliance (Dryer, Wash, Dish, Heat, Instant) over previous daily periods	∑_*ON*_{Appliance_*d−i*_,…,Appliance_*d*−*i*[+1]_}, *i* = 1,3,7
177–196	Number of switch on states (activations) for each appliance (Dryer, Wash, Dish, Heat, Instant) in previous i-day periods	∑_*ON*_{Appliance_*d−i*_,…,Appliance_*d*−*i*[+1]_}, *i* = 7,14,21,28,35
197–221	Number of hours between the most recent five successive activations of each device	∑_*G*_(Appliance_*ON*_, Appliance_*ON*[+1]_), *ON* = 0,…,5

The feature vector in Table 5 includes a number of switch on states (activations) collected for individual appliances: Clothes Dryer (Dryer), Clothes Washer (Wash), Dishwasher (Dish), Heat Pump (Heat), and Instant Hot Water Unit (Instant). The data report the appliances’ activations taking into account previous hours, previous days, previous weeks and the difference (in hours) between the most recent five successive activations (for each appliance).

The features presented in Table 5 are the outcome of segmentation and sequence analysis, and they describe, as widely as possible, the existing dependencies in the data. In particular, they revealed the following relations:

- The structures of the devices’ profiles over days, weeks and months, which were discovered by analyzing appliances’ switch on (activations) events in each hour (see Table 2 and S1 Table), and the outcome of the analysis of the contributions from variables no. 147 to 196, which are associated with the characteristics of a single device.
- The sequential patterns and the time periods between successive activations are reflected in variables no. 197 to 221 as a result of the sequence mining approach (see Paragraph 3.3).

### 4.3. An approach to forecasting

Building predictive models is a task that requires dealing with both huge data volumes and complex algorithms. Therefore, it becomes necessary to have an efficient computing environment with high-performance computers. In our case, all the numerical calculations were performed on computing clusters located at the Interdisciplinary Center for Mathematical and Computational Modelling at the University of Warsaw. The HYDRA engine with Scientific Linux 6 operating system was used with the following nodes and their parameters:

- *Istanbul*–AMD Opteron(tm) 2435 2.6 GHz, 2 CPU x 6 cores, 32 GB RAM.
- *Westmere*–Intel(R) Xeon(R) CPU X5660 2.8 GHz, 2 CPU x 6 cores, 24 GB RAM.

R-CRAN was used as the computing environment, which is an advanced statistical package, as well as an interpreted programming language that exists on Windows, Unix and MacOS platforms. It is licensed under the GNU GPL and based on the S language.

The starting point for the numerical experiments was the division of the AMPDs dataset into three parts, which corresponded to the training, validation and testing samples with the following proportions.

The training sample consisted of 330 days (7920 observations) between May 6th. 2012, and March 31st. 2013, the validation sample consisted of 28 days (672 observations) between April 1^st^. 2013, and April 28^th^. 2013, and finally, the testing sample consisted of 14 days (336 observations) between April 29^th^. 2013, and May 12^th^. 2013.

The main criterion taken into account while learning the models is to gain good generalization of knowledge with the least error. The most commonly used measure to assess the quality of forecasts in the electric power system is the MAPE. Therefore, to find the best parameters for all models and to assure their generalization, the following function was minimized:

$$f({MAPE}_{U},{MAPE}_{W})=\frac{1}{2}|{MAPE}_{U}-{MAPE}_{W}|+\frac{1}{2}{MAPE}_{W}.$$ (11)

where *MAPE*_*U*_ and *MAPE*_*W*_ stand for the training and validation errors, respectively.

In the experiments, the broad set of the machine learning algorithms was tested including artificial neural networks, regression trees, random forest regression, k-nearest neighbors regression and support vector regression. In the following discussion, the algorithms are briefly introduced along with their settings.

#### Artificial neural network

An artificial neural network (ANN) is a network that is often used to estimate or approximate functions that can depend on a large number of inputs. In contrast to other machine learning algorithms, they required special preparation of the input data. The vector of continuous variables has been standardized, while the binary variables were converted such that the value of 0 was transformed into -1. Finally, the dependent variable was normalized by zero unitarization. To propose the forecast, the reverse transformation of the dependent variable was applied.

To train the neural networks, we used the BFGS (Broyden–Fletcher–Goldfarb–Shanno) algorithm, which belongs to the broad family of quasi-Newton optimization methods (available in the *nnet* library). The network had an input layer with 146 and 221 variables, depending on whether the additional variables with usage patterns were considered in the model. In the hidden layer, a different number of neurons was tested, starting from 10 to 50 by 5 (nine sets).

A logistic function was used to activate all of the neurons in the network, and a regularization factor was introduced to penalize weights that were too high in the network (to control overfitting). Three different values of the factor were considered in the experiments: 0.01, 0.1 and 0.5.

Each time, 27 neural networks were learned with various parameters (the number of neurons in the hidden layer multiplied by the number of penalties). To avoid overfitting, after the completion of each learning iteration (with a maximum of 50 iterations), the models were checked for the error measure defined in Eq **(11)**.

At the end, out of 27 learned network, the one characterized by the smallest error defined in formula **(11)** was chosen as the best for delivering forecasts.

#### K-Nearest Neighbors regression

The K-Nearest Neighbors algorithm is a non-parametric method used for regression. The input consists of the k-closest training examples in the feature space, and the property of an object is obtained via a similar averaging process, where the value of the object is the average value of the k closest training points.

To improve the algorithm, we used the normalization of the explanatory variables (standardization for quantitative variables and replacement of 0 into -1 for the binary variables). The normalization assures that all dimensions for which the Euclidean distance is calculated have the same importance. Otherwise, it could lead to a situation in which a single dimension would dominate other dimensions.

The algorithm was trained with *knnreg* implemented in *caret* library. Different numbers of the k-nearest neighbors were proposed in the experiments including the following: {5, 10, 15, 20, 25, 30, 35, 40, 45, 50, 55, 60, 65, 70, 75, 80, 85, 90, 95, 100, 110, 120, 130, 140, 150, 160, 170, 180, 190, 200, 250, 300}.

As the optimal number of neighbors, and thus, the final form of the model was considered the one characterized by the smallest error defined in formula **(11)**.

#### Regression trees

To train regression trees, an *rpart* package that implements the CART algorithm was used. The criterion that was minimized in the process of dividing a multidimensional space was the dispersion (variance) around the mean value of the dependent variable for observations belonging to the same node (leaf). At each stage of the splitting node, the variable and its specific value that minimized the sum of squared was chosen. The minimum number of observations in the node was set to 20, and the leaf was set to at least 6 observations, otherwise the node was subject to splitting.

Instead of pruning the tree at the end of the algorithm, we used pruning during the growth of the tree. Generally, this approach will stop the process of creating new splits in case the previous splits provided only a slight increase in predictive accuracy. The complexity parameter (*cp*) was set from 0 to the value of 0.1 (with an increment value of 0.001), meaning that if any split does not increase the model’s overall coefficient of determination by at least *cp*, then the split is decreed to be, a priori, not worth pursuing. The tree was built up to a depth of 30 levels.

Out of 1.000 regression trees that were tested, the final structure was chosen, based on the error measure defined according to formula **(11)**.

#### Random regression forests

To train the random regression forest, an algorithm from the *randomForest* library was used.

Each time prior to the training, the n-element samples with replacement were drawn, and they accounted for approximately 63% of the population. The samples were used to construct the CART tree. Each tree has been built to its maximum size (without pruning), preventing the occurrence of 5 or fewer observations in a leaf.

A randomized subset of variables was used to construct each tree. The number of variables used in the models varied from 10 to 142 for the base model (without usage pattern variables) and from 10 to 217 for the enhanced model with variables describing the appliances’ usage patterns.

The total number of trees in the forest was 500. The final forecast was defined based on Huber’s robust estimator, as defined in [39].

Finally, as in previous cases, the best forest structure was chosen, based on the error measure defined in **(11).**

#### Support vector regression

To construct the support vector regression, the *ε-SVR* version was used from *kernlab* library with its SMO algorithm–Sequential Minimal Optimization to solve the quadratic programming problem. The linear function was used as a kernel function. The value of the ε–parameter, which defines the margin width for which the value of the error function is zero, was arbitrarily taken from the following set {0.01, 0.05, 0.1, 0.25, 0.5, 0.75, 1}.

The regularized parameter that controls the overfitting, C, was arbitrarily set, and the simulations were run for the following values {0.0001, 0.0005, 0.001, 0.005, 0.01, 0.05, 0.1, 0.25, 0.5, 0.75, 1}. Finally, as in all previous cases, based on the models’ results, the model that minimized function **(11)** was chosen.

The proposed machine learning algorithms were challenged against some typical approaches used for forecasting (benchmarks). Those were naive forecast, random forecast, the ARIMA model and stepwise regression.

#### Naive forecast

The naive forecast was constructed in the following manner: for the forecasting horizon of 24 hours, the value recorded on a previous day and at the respective hour was taken as the forecast.

#### Random forecast

The random forecast was constructed in the following way. Given the electricity consumption in a given hour with respect to the day of the week, empirical distribution functions were computed. Then, using a *runif* function, a value from a uniform distribution between 0 and 1 was drawn (*p* probability). This value was then used to estimate the quantile of empirical distribution (the final value of the forecast) by the weighted averaging of order statistics *z*_*g*_ (*quantile* function):

$$Q_{p}=(1-\gamma )z_{g}+\gamma z_{g+1}.$$ (12)

where *γ* = *np* + *m* − *g*, *n* is number of observations, *g* = *floor*(*np* + *m*) and *m* = 1 − *p*.

#### ARIMA model

The third method used to compare was the forecast developed with the *ARIMA*(*p*,*d*,*q*) model. The model was estimated using the *auto*.*arima* function implemented in the *forecast* library. The function identifies and estimates the model by minimizing the Akaike information criterion.

To estimate the model, the maximum values for the AR and MA order were arbitrary set to *p* = 14 and *q* = 14. The degree of differencing was tested with the *KPSS* test (Kwiatkowski–Phillips–Schmidt–Shin), which was used for testing a null hypothesis that an observable time series is stationary around a deterministic trend

#### Stepwise regression

The last method was stepwise linear regression as an automated tool used to identify a useful subset of predictors. Two procedures were tested: the one that adds the most significant variable (forward selection) and the second one that removes the least significant variable during each step (backward elimination). The forward approach stops when all variables not in the model have p-values that are greater than the specified alpha-to-enter value (5% significance level was used). The backward stops when all variables in the model have p-values that are less than or equal to the specified alpha-to-remove value (5% significance level was used).

### 4.4. The results of numerical experiments

Taking into account the clarity of the presented results, the following notations were introduced for the models: Z_24_ –naive forecast, Fg−random forecast, ARIMA–ARIMA model, L_f–stepwise regression with forward selection, L_b–stepwise regression with backward elimination, KNN–k-nearest neighbors regression, RPART–regression trees, RF–random regression forests, NNET–artificial neural network, and SVR–support vector regression.

The results of the models for the 24 hour forecasting horizon are presented in Table 6. For the testing sample, the MAPE varied from 56.27% for the random forecast (F_g_) to 26.78% for support vector regression (SVR). In terms of the r-MAPE, the highest error were observed for random forecast (F_g_)– 42.66%, while the lowest error was observed for support vector regression (SVR)– 25.26%, as previously observed. A small difference between the MAPE and the r-MAPE is observed, suggesting that the models do not exhibit very large errors in terms of both underestimation and overestimation.

**Table 6:** The average 24 hour forecasting results based on past usage data (without usage patterns variables).

Model	MAPE (%)	r_MAPE (%)	Acc (%)	MSE	MAPE (%)	r_MAPE (%)	Acc (%)	MSE	MAPE (%)	r_MAPE (%)	Acc (%)	MSE
	Training dataset				Validation dataset				Testing dataset			
Z_24_	42.94	33.79	40.90	0.61	40.33	31.58	43.68	0.59	37.69	29.78	45.83	0.44
F_g_	51.86	41.66	34.28	0.72	45.74	37.89	37.59	0.77	56.27	42.66	37.80	0.63
ARIMA	38.24	35.27	31.01	0.35	50.60	49.34	18.57	0.49	41.14	39.39	20.83	0.26
L_f	36.08	33.67	34.37	0.32	33.72	32.08	35.81	0.43	36.50	34.25	35.71	0.23
L_b	36.03	33.64	34.51	0.32	34.19	32.37	35.07	0.40	36.05	34.07	33.63	0.22
KNN	33.36	31.42	36.15	0.31	35.01	33.67	33.28	0.39	30.27	28.89	41.37	0.22
RPART	37.08	33.66	36.36	0.32	38.69	35.33	37.00	0.42	38.50	34.20	35.12	0.24
RF	0.48	0.34	100.00	0.00	32.72	30.86	37.89	0.39	32.57	29.74	41.07	0.22
NNET	32.39	30.11	39.99	0.34	32.19	30.06	41.60	0.41	30.28	28.14	42.26	0.21
SVR	28.73	27.26	43.98	0.35	28.95	28.06	44.43	0.46	26.78	25.26	47.02	0.23

The precision of how close the model is able to forecast to the actual load (MSE) revealed that artificial neural networks (NNET) are able to forecast with the least error– 0.21. In contrast, the random forecast (F_g_) had an MSE of 0.63.

In the case of the accuracy (Acc), which measures how many correct forecasts the model makes, it was observed that the highest accuracy was achieved with support vector regression (SVR)– 47.02%. Surprisingly, the second best result was observed for the naive forecast (Z_24_)– 45.83%. The least precise forecast was obtained with the ARIMA model–an accuracy of only 20.83%.

In general, the proposed machine learning methods (except regression trees–RPART) in comparison to benchmarking methods had smaller MSE, MAPE, and r-MAPE and higher accuracy rates.

The same techniques were applied to the enhanced dataset including the behavioral variables. The results are summarized in Table 7.

**Table 7:** The average 24 hour forecasting results based on past usage data and enhanced data with usage pattern variables.

Model	MAPE (%)	r_MAPE (%)	Acc (%)	MSE	MAPE (%)	r_MAPE (%)	Acc (%)	MSE	MAPE (%)	r_MAPE (%)	Acc (%)	MSE
	Training dataset				Validation dataset				Testing dataset			
Z_24_	42.94	33.79	40.90	0.61	40.33	31.58	43.68	0.59	37.69	29.78	45.83	0.44
F_g_	51.86	41.66	34.28	0.72	45.74	37.89	37.59	0.77	56.27	42.66	37.80	0.63
ARIMA	38.24	35.27	31.01	0.35	50.60	49.34	18.57	0.49	41.14	39.39	20.83	0.26
L_f	36.08	33.59	34.29	0.32	35.92	34.26	30.01	0.42	43.11	41.37	30.06	0.25
L_b	35.93	33.40	34.86	0.32	36.56	34.32	32.24	0.40	33.26	31.30	38.69	0.22
KNN	38.50	36.99	27.17	0.35	41.12	39.02	27.19	0.40	34.00	32.44	33.93	0.22
RPART	39.18	35.61	35.16	0.36	39.86	35.87	35.36	0.42	39.38	36.03	38.39	0.23
RF	0.48	0.36	100.00	0.00	33.26	31.13	37.15	0.39	29.41	27.33	48.21	0.21
NNET	29.65	28.10	42.05	0.36	27.61	26.28	46.21	0.45	23.62	22.54	54.46	0.25
SVR	32.94	31.53	34.67	0.34	32.39	31.39	35.51	0.46	40.49	38.80	23.51	0.24

To identify situations in which additional explanatory (behavioral) variables describing electricity usage patterns improved the final forecast, we introduced a percentage point sensitivity range, denoted with the following colors:

- green shows forecast improvements in terms of Acc and MAPE/r-MAPE when building the model using the enhanced features dataset (with usage pattern variables), e.g., for the model that has 20% error, the improvement should be at least 0.5 p.p. so the model’s error should be less than 19.5%;
- red shows forecast worsening in terms of Acc and MAPE/r-MAPE when building the model using the enhanced features dataset (with usage pattern variables), e.g., for the model that has 20% error, the error increase should be at least 0.5 p.p. so the model’s error should be greater than 20.5%;
- no color shows neutral cases in which Acc and MAPE/r-MAPE stayed at similar levels, e.g., for the model that has 20% error, we define 1 p.p. range (19.5%–20.5%) to say that no improvement is observed when building the model using enhanced features dataset (with usage pattern variables).

In comparison to the modeling without behavioral pattern variables, the results associated with the testing dataset are better for two machine learning algorithms, i.e., the random forests and artificial neural networks, and for the stepwise regression model. In the case of the artificial neural network model, the improvement was substantial: the MAPE decreased by 6.66 p.p. (23.62%), r-MAPE decreased by 5.6 p.p. (22.54%), and Acc was improved by 12.2 p.p. (54.46%). Moreover, this model has the best results among all of the forecasting methods.

For the random regression forests, the forecast was improved by 3.16 p.p. (29.41%) in terms of MAPE, by 2.41 p.p. (27.33%) in terms of r-MAPE, and finally, the accuracy was improved by 7.14 p.p. (48.21%).

For the stepwise regression model with backward selection, the MAPE decreased by 2.79 p.p. (33.26%), r-MAPE decreased by 2.77 p.p. (31.30%), and accuracy was improved by 5.06 p.p. (38.69%).

**Fig 7:**
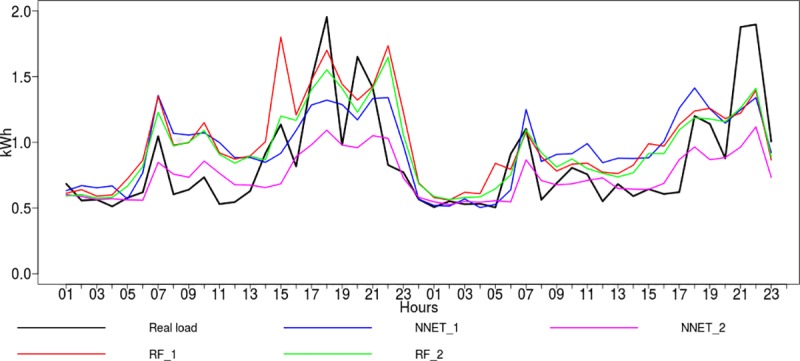
The real load vs. forecasts of the neural network and random forest models.

The two techniques with the most accurate forecasts are presented in graphical form in Fig 7. For this reason, the real value and the forecasts provided by the neural networks and random forests were presented for two randomly selected test days–May 7th and 8th. 2013.

The following notations were used: NNET_1 and RF_1 indicate forecasts prepared using the dataset with past electricity consumption variables only, NNET_2 and RF_2 indicate forecasts build on the enhanced dataset including the household behavioral data.

For the neural networks (NNET_1) and the random forest regression (RF_1), which were trained on the dataset with a limited number of variables, it was observed that the forecasts were higher than the actual load value. Adding behavioral variables resulted in decreases in the error. However, overestimation is eliminated, but at the same time, there are some problems with underestimation. In particular, this is observed when relatively large energy loads occur.

In general, from the figure we can observe how the forecasts follow the real load curve. The trend is followed well enough, but as expected, due to household behavior and other immeasurable influences, there are some deviations when comparing the forecasts and the real load.

## 5. Scalability of the approach

To draw meaningful conclusions and to provide rationale for the proposed approach, additional experiments were undertaken. For this purpose, the numerical analyses were performed for the group of 46 households based on the WikiEnergy data [7]. This enabled us to formulate generalized conclusions about the modeling techniques and household specific behavioral data in terms of the applicability of both for the benefit of accurate forecasting modeling.

The WikiEnergy dataset by Pecan Street Inc. is a large database of consumer energy information. This database is highly granular, including the usage measurements collected from up to 24 circuits within the home. The investigated households were located in Austin, Texas, USA. From these data, we have extracted 14 months of data from 46 households at a granularity of 1 hour, covering the same time window from March 2013 until April 2014.

The dataset was split into three parts, which corresponded to the training, validation and testing samples with the following proportion. The training sample consisted of 330 days (7920 observations) between April 5th. 2013, and February 28th. 2014; the validation sample consisted of 28 days (672 observations) between March 1^st^. 2014, and March 28^th^. 2014; and finally, the testing sample consisted of 14 days (336 observations) between March 29^th^. 2014, and April 11^th^. 2014.

The same feature vectors describing basic electricity consumption and an enhanced dataset with usage patterns were prepared, and the same techniques were trained, as previously for AMPDs data.

The aggregated results for all 46 households are presented in Table 8. This is to check whether the enhanced dataset, including the usage patterns, improved the forecasts in terms of the MAPE observed on the test dataset for each of the modeling methods. The main finding is that the neural networks forecasts prepared on the enhanced dataset (including usage patterns) were more accurate than those prepared using the basic data set with only historical usage variables.

**Table 8:** Aggregated results in terms of the MAPE for 46 households. The number of households is presented in brackets.

Results	Modeling method						
	L_f	L_b	KNN	RPART	RF	NNET	SVR
**Improving**	23.91% (11)	10.87% (5)	36.96% (17)	39.13% (18)	15.22% (7)	82.61% (38)	19.57% (9)
**Worsening**	71.74% (33)	84.78% (39)	56.52% (26)	26.09% (12)	71.74% (33)	6.52% (3)	69.57% (32)
**Neutral**	4.35% (2)	4.35% (2)	6.52% (3)	34.78% (16)	13.04% (6)	10.87% (5)	10.87% (5)
**Sum**	100.00% (46)	100.00% (46)	100.00% (46)	100.00% (46)	100.00% (46)	100.00% (46)	100.00% (46)

The neural network outperformed the other methods in terms of MAPE. The forecast proposed for 82.61% of the households (that is 38 households out of 46) had the lowest error, RPART was the second most accurate forecasting method, which delivered accurate forecasts for 39.13% of the households (that is 18 households out of 46).

**Fig 8:**
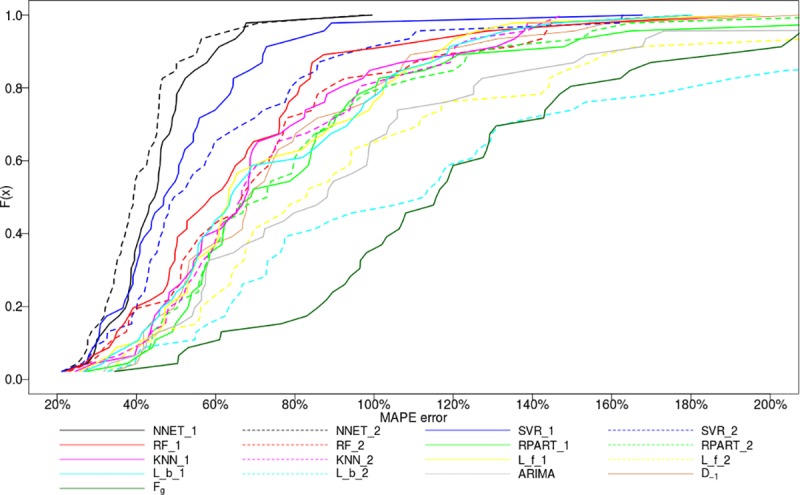
Modelling techniques and their MAPE distributions observed on the testing dataset.

The MAPE distributions were also analyzed as presented in Fig 8. The forecasts prepared based on the dataset with past electricity consumption variables only are shown with the solid line (e.g., NNET_1, SVR_1). The forecasts build on the enhanced datasets including household behavioral data are presented with dotted lines (e.g., NNET_2 and SVR_2). In general, it could be observed that the machine learning models exhibit smaller errors than the benchmarking methods.

Finally, to provide a quantitative summary of the experiments, we tested the following:

1 Whether there are statistically significant differences in terms of the errors between the forecasting algorithms, including the machine learning algorithms and the benchmarking methods–please refer to Table 9 for details. The Duncan multiple range test (DMRT) was used to compare differences among the error means between the modeling techniques and across datasets (basic consumption variables vs. enhanced dataset). Differences were found to be significant at P < 0.05.

**Table 9:** Duncan's Multiple Range Test to assess the differences between the forecasting algorithms. The different capital letters within each column are significantly different (P < 0.05).

Modelling method	Basic consumption dataset	Enhanced dataset including usage patterns
**Z**_**24**_	A	A
**Fg**	A	B
**ARIMA**	B	BC
**L_f**	BC	BCD
**L_b**	BC	BCDE
**KNN**	BC	BCDE
**RPART**	BCD	BCDE
**RF**	BCD	CDE
**NNET**	CD	DE
**SVR**	D	E

2 Whether there are statistically significant differences in terms of the MAPE for the neural networks (as the most accurate forecasting technique) with respect to the data, i.e., the basic dataset vs. the enhanced dataset with household activity patterns. Please refer to Table 10 for details. The Kolmogorov-Smirnov (K-S) test was used to compare two empirical error distributions with respect to the data sets. The differences were found significant at P < 0.05.

**Table 10:** The Kolmogorov-Smirnov (K-S) test to determine the significance of the forecasting results between the basic dataset and the enhanced dataset using the neural networks method, * denotes significance at P < 0.05.

Household	Basic consumption dataset MAPE (%)	Enhanced dataset MAPE (%)	K-S p-value	Household	Basic consumption dataset MAPE (%)	Enhanced dataset MAPE (%)	K-S p-value
1	46.07	36.13	0.039 *	24	29.11	31.89	0.864
2	52.33	44.56	0.008 *	25	33.18	30.51	0.097
3	99.46	97.68	0 *	26	35.61	32.80	0.269
4	21.43	21.28	0.381	27	49.08	45.74	0 *
5	67.71	39.69	0.000 *	28	39.48	38.04	0.423
6	59.28	56.43	0.026 *	29	37.98	35.13	0.114
7	37.21	32.15	0.269	30	44.62	45.13	0.269
8	45.20	39.14	0.207	31	60.89	50.11	0.304
9	45.96	45.65	0.511	32	46.18	46.10	0.651
10	40.13	39.24	0.010 *	33	50.08	45.45	0.180
11	67.01	63.61	0.097	34	48.89	46.55	0.155
12	30.00	28.61	0.341	35	42.95	33.53	0 *
13	48.33	45.27	0.010 *	36	40.78	38.28	0.269
14	45.61	34.34	0.001 *	37	29.74	26.71	0.01 *
15	43.29	39.54	0.03 *	38	42.08	37.00	0.047 *
16	57.77	55.06	0.03 *	39	45.54	70.94	0.864
17	50.16	40.31	0 *	40	65.02	55.70	0 *
18	31.67	27.67	0.001 *	41	51.30	45.85	0.003 *
19	41.24	42.89	0.097	42	38.69	36.28	0.207
20	38.62	37.49	0.697	43	39.54	27.72	0 *
21	49.86	43.26	0.026 *	44	27.60	25.13	0 *
22	54.68	50.08	0.014 *	45	46.61	42.56	0.133
23	38.51	34.87	0.017 *	46	37.91	34.30	0.068

The results presented in Table 9 indicate that there are significant differences among the forecasting results from the machine learning techniques (capital letter D), namely, SVR, NNET, RF, RPART and the benchmarking methods (capital letter A), namely, Z_24_ and Fg, observed when using the basic consumption dataset. No differences are observed between the results provided by L_f, L_b, KNN, RPART, RF and NNET (capital letter C). For the enhanced dataset, the differences are less diverse, e.g., the NNET results are significantly different from Z_24_, Fg and the ARIMA results and, at the same time, not significantly different from those obtained with RF, RPART, KNN, L_B, and L_f (capital letter D). The analysis to determine the significance of the forecasting results between the basic dataset vs. the enhanced dataset for the neural networks (as the most accurate forecasting technique) revealed that a significant difference is observed for 50% of the households, although an improvement of at least 1 p.p. in terms of the MAPE was observed for 82.61% of the households (in green), as presented in Table 10. In three cases, the forecasts prepared using the enhanced dataset resulted in less accurate forecasts (in red); however, the differences were not significant. The richer dataset helped to reduce the MAPE by 8.5% on average, that is, from 45.5% to 41.7%, calculated over the whole population of 46 households.

## 6. Conclusions

In this paper, we presented an extensive analysis aimed at forecasting electricity loads on the individual household level, which potentially brings greater intelligence to smart meters and delivers value added for individual customers.

The experiments were designed to find answers to research questions concerning the forecasting loads for individual customers. In particular, the findings are as follows:

1. Based on the results, we can conclude that it **is possible to provide accurate load forecasting for 24 hours ahead on the individual household level, and this can be obtained with reasonable prediction accuracy.** The forecasts of the neural network models show that they have good performance characterized by low errors obtained on both datasets, i.e., a basic one with past usage data and a richer dataset with usage patterns. Based on the AMPDs data, the richer dataset helped to reduce the MAPE from 30.28% to 23.62% and, on average, from 45.5% to 41.7% for the WikiEnergy data.
2. **The clustering and sequence recognition algorithms are good tools for identifying patterns of household behavior.** They allowed quickly grasping general trends in data and then clustering appliances based on their typical usage hours. The data obtained by grade analysis might be the basis for the decision support for individual customers to consider the price elasticity tariffs and for accurate forecasting in smart metering applications. Sequence analysis gave insight that can help understand how power consumption is influenced by certain activities and their sequences and how those activities are related to each other.
3. We showed through experiments that a **combination of historical usage data and household behavioral data can greatly enhance the forecasting of individual consumer loads**. The richer data set can reduce MAPE by 8.5% on average and up to 41% for individual households (e.g., household #5 from the WikiEnergy dataset) as observed on the test set for the neural network model.
4. The results indicate that there are significant differences in forecasting in favor of the machine learning techniques, namely, SVR, NNET, RF, and RPART, in comparison to random forecasts or ARIMA models. In particular, **artificial neural networks** through their hidden layers and ability to approximate complex nonlinear mappings directly from the input samples **seem to be very effective at solving short-term forecasting when dealing with high volatility data**. They are able to identify hidden trends and make use of richer data, thereby finding the trends in household consumption data.

As a future work, we will explore algorithmic approaches for mining typical usage patterns and utilize them for the purpose of energy consumption forecasting and the development of unique, individualized energy management strategies. Additionally, considerable interest and high expectations worldwide are associated with attempts to combine research on forecasting systems utilizing non-intrusive appliance recognition and user patterns with multi agent systems [40–42]. Such a multi-agent computer system can be used for managing the unbalanced energy in a microgrid, and the main goal of the system would be to control and minimize the differences between the current energy demand and the actual energy supply.

## Supporting information

S1 Table The matrix with the probabilities of appliance turn ON events in each hour over whole week. (XLSX)

## References

[1] Anvari-Moghaddam A. Monsef H. Rahimi-Kian A. Optimal smart home energy management considering energy saving and a comfortable lifestyle. IEEE Transactions on Smart Grid. 2015; 6(1): 324–332.

[2] Zhou B. Li W. Chan K.W. Cao Y. Kuang Y. Liu X. Wang X. Smart home energy management systems: concept, configurations, and scheduling strategies. Renewable and Sustainable Energy Reviews. 2016; 61: 30–40.

[3] Wu X. Hu X. Moura S. Yin X. Pickert V. Stochastic control of smart home energy management with plug-in electric vehicle battery energy storage and photovoltaic array. Journal of Power Sources. 2016; 333: 203–212.

[4] Hu X. Murgovski N. Johannesson L. Egardt B. Optimal dimensioning and power management of a fuel cell/battery hybrid bus via convex programming, IEEE/ASME Transactions on Mechatronics. 2015; 20(1): 457–468.

[5] Sun C. Sun F. Moura S. Nonlinear predictive energy management of residential buildings with photovoltaics & batteries. Journal of Power Sources. 2016; 325: 723–731.

[6] Makonin S. Popowich F. Bartram L. Gill B. Bajic IV. AMPds: A public dataset for load disaggregation and eco-feedback research. IEEE Electrical Power & Energy Conference (EPEC). 2013; pp. 1–6.

[7] Pecanstreet. Dataport. 2014. Available from https://dataport.pecan.street.org/

[8] Hong T. Pinson P. Fan S. Global energy forecasting competition 2012. International Journal of Forecasting. 2014; 30(2): 357–363.

[9] Beaudin M. Zareipour H. Home energy management systems: A review of modelling and complexity. Renewable and Sustainable Energy Reviews. 2015; 45: 318–335.

[10] Gross G. Galiana FD. Short-term load forecasting. Proceedings of the IEEE. 1978; 75(12):1558–1573.

[11] Weron R. Modeling and Forecasting Electricity Loads and Prices: A Statistical Approach. Chichester: Wiley; 2006.

[12] Brockwell PJ. Davis RA. Introduction to Time Series and Forecasting. 2nd ed. Heidelberg: Springer; 2002.

[13] Song KB. Baek YS. Hong DH. Jang G. Short-term load forecasting for the holidays using fuzzy linear regression method. IEEE Transactions on Power Systems. 2005; 20: 96–101.

[14] Szupiluk R. Wojewnik P. Ząbkowski T. Prediction improvement via smooth component analysis and neural network mixing In: Kollias SD. Stafylopatis A Duch W. Oja E. editors. Lecture Notes in Computer Science. Heidelberg: Springer; 2006b; 4132: 133–140.

[15] Siwek K. Osowski S. Szupiluk R. Ensemble neural network approach for accurate load forecasting in a power system. International Journal of Applied Mathematics and Computer Science. 2009; 19(2): 303–315.

[16] Beccali M. Cellura M. Brano VL. Marvuglia A. Forecasting daily urban electric load profiles using artificial neural networks. Energy Conversion and Management. 2004; 45: 2879–2900.

[17] Suna C. Suna F. Mourab S.J. Nonlinear predictive energy management of residential buildings with photovoltaics & batteries. Journal of Power Sources. 2016; 325: 723–731.

[18] Lv G. Wang X. Jin Y. Short-Term Load Forecasting in Power System Using Least Squares Support Vector Machine. Proceedings of the 2006 International Conference on Computational Intelligence. Theory and Applications. 2006; pp. 117–126.

[19] Giorgi de MG. Campilongo S. Ficarella A. Congedo PM. Comparison Between Wind Power Prediction Models Based on Wavelet Decomposition with Least-Squares Support Vector Machine (LS-SVM) and Artificial Neural Network (ANN). Energies. 2014; 7: 5251–5272.

[20] Siwek K. Osowski S. Szupiluk R. Wojewnik P. Ząbkowski T. Blind Source Separation for Improved Load Forecasting in the Power System. IEEE Proceedings of the European Conference on Circuit Theory and Design. 2005; pp. 61–64.

[21] Szupiluk R. Wojewnik P. Ząbkowski T. Combining Forecasts with Blind Signal Separation Methods in Electric Load Prediction Framework. Proceedings of 24th IASTED Artificial Intelligence and Applications. AIA. 2006a; pp. 439–444.

[22] Ghofrani M. Hassanzadeh M. Etezadi-Amoli M. Fadali MS. Smart meter based short-term load forecasting for residential customers. North American Power Symposium (NAPS). 2011; pp. 1–5.

[23] Gajowniczek K. Ząbkowski T. Data Mining Techniques for Detecting Household Characteristics Based on Smart Meter Data. Energies. 2015a; 8(7): 7407–7427.

[24] Gajowniczek K. Ząbkowski T. Short term electricity forecasting based on user behavior from individual smart meter data. Journal of Intelligent and Fuzzy Systems. 2015b; 30(1): 223–234.

[25] Javed F. Arshad N. Wallin F. Vassileva I. Dahlquist E. Forecasting for demand response in smart grids: an analysis on use of anthropologic and structural data and short term multiple loads forecasting. Applied Energy. 2012; 69: 151–160.

[26] Edwards RE. New J. Parker LE. Predicting future hourly residential electrical consumption: A machine learning case study. Energy and Buildings. 2012; 49: 591–603.

[27] Ziekow H. Goebel C. Struker J. Jacobsen H. The potential of smart home sensors in forecasting household electricity demand. IEEE International Conference on Smart Grid Communications. 2013.

[28] Singh RP. Gao PX. Lizotte DJ. On hourly home peak load prediction. IEEE International Conference on Smart Grid Communications. 2013; pp. 163–168.

[29] Ding Y. Borges J. Neumann MA. Beigl M. Sequential pattern mining–A study to understand daily activity patterns for load forecasting enhancement. IEEE First International Conference on Smart Cities. 2015; pp. 1–6.

[30] Veit A. Goebel C. Tidke R. Doblander C. Jacobsen HA. Household electricity demand forecasting: benchmarking state-of-the-art methods. Proceedings of the 5th International Conference on Future Energy Systems. 2014; pp. 233–234.

[31] Ward JH. Hierarchical grouping to optimize an objective function. J. Am. Stat. Assoc. 1963; 58: 236–244.

[32] Szczesny W. On the performance of a discriminant function. Journal of Classification.1991; 8: 201–215.

[33] GradeStat. Program for Grade Data Analysis. 2004. Available from http://gradestat.ipipan.waw.pl/

[34] Ciok A. Kowalczyk T. Pleszczyńska E. How a New Statistical Infrastructure Induced a New Computing Trend in Data Analysis. Polkowski L. Skowron A. editors. Rough Sets and Current Trends in Computing. Lecture Notes in Artificial Intelligence. Heidelberg: Springer; 1998; 1424: 75–82.

[35] Ciok A. Kowalczyk T. Pleszczyńska E. Szczesny W. Algorithms of grade correspondence-cluster analysis. Archiwum Informatyki Teoretycznej i Stosowanej 1995; 7: 5–22.

[36] Giudici P. Applied Data Mining Statistical Methods for Business and Industry. Chichester: Wiley; 2003.

[37] Rao CR. Wegman EJ. Solka JL. Handbook of Statistics. Data Mining and Data Visualization. 2005; 24: 1–644.

[38] Moreno JJM. Pol AP. Abad AS. Blasco BC. Using the R-MAPE index as a resistant measure of forecast accuracy. Psicothema. 2013; 25(4): 500–506. doi: 10.7334/psicothema2013.23 24124784 10.7334/psicothema2013.23

[39] Huber PJ. Robust Statistics. New York: Wiley; 1981.

[40] Radziszewska W. Kowalczyk R. Nahorski Z. El Farol Bar problem. Potluck problem and electric energy balancing–on the importance of communication. Proceedings of the 2014 Federated Conference on Computer Science and Information Systems. 2014a; pp. 1515–1523.

[41] Radziszewska W. Nahorski Z. Parol M. Pałka P. Intelligent computations in an agent-based prosumer-type electric microgrid control system. Issues and Challenges of Intelligent Systems and Computational Intelligence. 2014b; pp. 293–312.

[42] Pałka P. Radziszewska W. Nahorski Z. Balancing electric power in a microgrid via programmable agents auctions. Control and Cybernetics. 2012; 41(4): 777–797.

